# Apelin Increases Cardiac Contractility via Protein Kinase Cε- and Extracellular Signal-Regulated Kinase-Dependent Mechanisms

**DOI:** 10.1371/journal.pone.0093473

**Published:** 2014-04-02

**Authors:** Ábel Perjés, Réka Skoumal, Olli Tenhunen, Attila Kónyi, Mihály Simon, Iván G. Horváth, Risto Kerkelä, Heikki Ruskoaho, István Szokodi

**Affiliations:** 1 Department of Pharmacology and Toxicology, Institute of Biomedicine, University of Oulu, Oulu, Finland; 2 Heart Institute, Medical School, University of Pécs, Pécs, Hungary; 3 Szentágothai Research Center, University of Pécs, Pécs, Hungary; 4 Faculty of Pharmacy, University of Helsinki, Helsinki, Finland; 5 Medical Research Center Oulu, Oulu, Finland; Max-Delbrück Center for Molecular Medicine (MDC), Germany

## Abstract

**Background:**

Apelin, the endogenous ligand for the G protein-coupled apelin receptor, is an important regulator of the cardiovascular homoeostasis. We previously demonstrated that apelin is one of the most potent endogenous stimulators of cardiac contractility; however, its underlying signaling mechanisms remain largely elusive. In this study we characterized the contribution of protein kinase C (PKC), extracellular signal-regulated kinase 1/2 (ERK1/2) and myosin light chain kinase (MLCK) to the positive inotropic effect of apelin.

**Methods and Results:**

In isolated perfused rat hearts, apelin increased contractility in association with activation of prosurvival kinases PKC and ERK1/2. Apelin induced a transient increase in the translocation of PKCε, but not PKCα, from the cytosol to the particulate fraction, and a sustained increase in the phosphorylation of ERK1/2 in the left ventricle. Suppression of ERK1/2 activation diminished the apelin-induced increase in contractility. Although pharmacological inhibition of PKC attenuated the inotropic response to apelin, it had no effect on ERK1/2 phosphorylation. Moreover, the apelin-induced positive inotropic effect was significantly decreased by inhibition of MLCK, a kinase that increases myofilament Ca^2+^ sensitivity.

**Conclusions:**

Apelin increases cardiac contractility through parallel and independent activation of PKCε and ERK1/2 signaling in the adult rat heart. Additionally MLCK activation represents a downstream mechanism in apelin signaling. Our data suggest that, in addition to their role in cytoprotection, modest activation of PKCε and ERK1/2 signaling improve contractile function, therefore these pathways represent attractive possible targets in the treatment of heart failure.

## Introduction

Apelin is the endogenous ligand of the former ‘orphan’ APJ receptor, a G-protein-coupled receptor (GPCR), now known as the apelin receptor (gene symbol *APLNR*) [1], [2] (reviewed in [3]). The apelin–apelin receptor signaling axis is emerging as an important regulator of the cardiovascular homoeostasis. Given a wide array of putative beneficial effects of apelin in various conditions, such as atherosclerosis [4], myocardial infarction [5], heart failure [6]–[9], arrhythmias [10] and pulmonary arterial hypertension [11], the apelin–apelin receptor system represents a promising therapeutic target in cardiovascular disease states. The peptide offers numerous potential advantages, particularly for heart failure therapy: apelin is a potent vasodilator of arteries [7], [12] and veins [12], [13] thereby reducing pre- and afterload, antagonizes the renin-angiotensin system [4], has anti-fibrotic effects [14], and enhances the force of cardiac contractions [15]–[17]. It is especially interesting that the positive inotropic effect of the peptide is preserved in the failing heart [6], [16], [17], although the apelin–apelin receptor system is chronically downregulated in heart failure [18], [19].

Apelin has been identified as one of the most potent endogenous stimulators of cardiac contractility [15]. Previously, we have demonstrated that the apelin-induced positive inotropic effect was partially suppressed by pertussis toxin (PTX), and inhibition of phospholipase C (PLC) and protein kinase C (PKC), suggesting that the peptide may enhance cardiac contractility via both PTX-sensitive Gα_i_ and -insensitive Gα_q_ proteins [15]. However, the downstream mechanisms mediating the inotropic effect of apelin are not fully understood. The positive inotropic response to various Gα_q_-coupled receptor agonists, such as endothelin-1 (ET-1) and phenylephrine, occur mainly via myofilament Ca^2+^ sensitization [20]. Early data suggested that ET-1 may stimulate cardiac contractility via PKC-dependent activation of Na^+^-H^+^ exchanger (NHE) with subsequent intracellular alkalization [21]. On the contrary, more recent evidence demonstrated that PKC-mediated positive inotropic response was not associated with alteration of intracellular pH [22]. It is possible, however, that PKC activation induces cardiac contractility by enhancing phosphorylation of sarcomeric proteins, including myosin regulatory light chain (RLC) [23] and troponin I (TnI) [20]. Other studies suggested that cardiac myosin light chain kinase (MLCK), but not PKC, is the predominant kinase for ventricular RLC [24]. Our former observations indicate that apelin elicits its positive inotropic effect primarily through increasing the sensitivity of myofilaments to Ca^2+^ rather than increasing intracellular Ca^2+^ transients [17]. In vascular smooth muscle cells, apelin has been reported to increase RLC phosphorylation [25]. However, it is not known whether apelin enhances cardiac contractility by increasing RLC phosphorylation in either a PKC- or an MLCK-dependent manner. Recently, we have demonstrated that the mitogen-activated protein kinases (MAPKs), extracellular signal-regulated kinase 1/2 (ERK1/2) and p38-MAPK, are major regulators of cardiac contractility [26]. Previous studies showed that apelin can enhance ERK1/2 signaling in various cell types [5], [8], [27], [28]; however, no information is available on whether MAPKs are involved in modulating the inotropic response to apelin. The objective of the present study was to characterize the role of PKC, MLCK, ERK1/2 and p38-MAPK in the regulation of cardiac contractility stimulated by apelin in the intact adult rat heart.

## Materials and Methods

### Ethical Statement

All protocols were reviewed and approved by the Animal Use and Care Committee of the University of Oulu.

### Materials

Drugs used were Apelin-16 (Phoenix Europe GmbH, Karlsruhe, Germany); Bisindolylmaleimide I (Bis) (Merck Chemicals Ltd., Nottingham, UK); ML-7 (Sigma-Aldrich, St. Louis, MO, USA); U0126 (LC Laboratories, Woburn, MA, USA). Apelin was dissolved in 0.6% acetic acid; Bis, ML-7 and U0126 were dissolved in dimethyl sulfoxide (DMSO). The final DMSO concentration was <0.15% in the perfusion buffer. DMSO and acetic acid were added to each vehicle control experiments in volumes equal to those used for diluting the drugs in parallel experiments.

### Isolated Perfused Rat Heart Preparation

Male 7-week-old Sprague-Dawley rats from the Center for Experimental Animals at the University of Oulu were used (n = 134). Rats were decapitated and hearts were quickly removed and arranged for retrograde perfusion by the Langendorff technique as described previously [21]. The hearts were perfused with a modified Krebs-Henseleit bicarbonate buffer, pH 7.40, equilibrated with a mixture of 95% O_2_ and 5% CO_2_ at 37°C. Hearts were perfused at a constant flow rate of 5.5 mL/min with a peristaltic pump (Minipuls 3, model 312, Gilson, Villiers, France). Heart rate was maintained constant (305±1 beats per minute) by atrial pacing using a Grass stimulator (model S88, Grass Instruments, West Warwick, RI, USA) (11 V, 0.5 ms). Contractile force (apicobasal displacement) was obtained by connecting a force displacement transducer (FT03, Grass Instruments, West Warwick, RI, USA) to the apex of the heart at an initial preload stretch of 20 mN. Perfusion pressure reflecting coronary vascular resistance was measured by a pressure transducer (model BP-100, iWorx Systems, Inc., Dover, NH, USA) situated on a side arm of the aortic cannula. Data were recorded using IX-228 Data Acquisition System and LabScribe recording and analysis software (iWorx Systems, Inc., Dover, NH, USA).

### Experimental Design

The experiments started with an equilibration period (40±4 min) and a 5-minute control period. Then various drugs were added to the perfusate for 5, 10, 15 or 20 minutes. Drugs used were apelin-16 (apelin); Bis; ML-7 and U0126. The doses of apelin were determined by pilots before each set of experiments to achieve comparable inotropic responses to that of our previous study [15]. After the end of experiments, hearts were rapidly dissected, left ventricular (LV) samples were frozen in liquid nitrogen and they were stored in –70°C until assayed.

### Western Blot Analysis for PKC Isoform Translocation

We followed the protocol found to be the most effective in preserving PKC isoforms by Hunter and Korzick [29] for sample preparation. Briefly, frozen LV tissues were grinded in liquid nitrogen and were dissolved and homogenized in ice-cold lysis buffer (20 mmol/L Tris, pH 7.5; 2 mmol/L each of EDTA and EGTA, pH 7.5–8.0; 5 mmol/L sodium fluoride; 5 μg/ml each of leupeptin and aprotinin; 0.5 μg/ml pepstatin A; 0.3 mmol/L phenylmethylsulfonyl fluoride; 1 AM vanadate; 3 mmol/L dithiothreitol) using a glass-glass tissue grinder. Samples were then centrifuged at 100 000×g for 1 h at 4°C, and the supernatant was defined as the soluble fraction. Pellets were resuspended in ice-cold lysis buffer containing 1% TritonX for 30 min. This fraction was then cleared with a 1 h 100 000×g centrifugation (4°C), and the resulting supernatant was defined as the particulate fraction. Protein concentrations were determined by the method of Bradford. Protein extracts were matched for protein concentration and stored denaturated in SDS loading buffer at −70°C. Equal protein volumes (7 μg) of particulate and soluble fraction were loaded onto conventional 7.5% SDS-polyacrylamide gels and transferred to nitrocellulose membranes. The membranes were blocked in 50% Odyssey blocking buffer (LI-COR GmbH, Bad Homburg, Germany) in TBS-tween and incubated with indicated primary antibody overnight. Protein levels were detected using fluorescence by Odyssey CLx infrared imaging System (LI-COR GmbH, Bad Homburg, Germany). Isoform-specific anti-PKCα antibody was from Sigma (Saint Louis, MO, USA) and anti-PKCε antibody was from Santa Cruz Biotechnology, Inc. (Dallas, TX, USA).

### Western Blot Analysis for RLC Phosphorylation

LV samples were subjected to urea/glycerol poliacrilamide gel electrophoresis (PAGE) to separate phosphorylated and nonphosphorylated RLC as described previously [24], [30]. We used a slightly modified protocol of Hidalgo et al. for protein isolation. Briefly, frozen LV tissues were grinded in liquid nitrogen and were dissolved in freshly prepared ice-cold urea sample buffer (9 mol/L urea, 50 mmol/L Tris, 300 mmol/L glycine, 5 mmol/L dithiothreitol, and 0.001% bromophenol blue, pH 8.6. containing of 50% glycerol and 1∶100 Protease inhibitor cocktail and Phosphatase Inhibitor cocktail 3 (Sigma-Aldrich, St. Louis, MO, USA)). Then the samples were transferred to a water bath at 60°C and were shaken there for 4 min. Samples were then centrifuged at 1300×g for 5 min at 4°C. The supernatant was collected and protein concentrations were determined by the method of Bradford. Samples were set to standard concentration by addition of lysis buffer and were stored at −70°C. The mobility of proteins in the non-denaturating urea glycerol PAGE varies with the electrical charge of the protein, phosphorylation and diphosphorylation result in additional migration of RLC in the urea-PAGE system, and thus the different grades of RLC-phosphorylation are represented by separate bands in the gel. Equal volumes (7 μg) of proteins were loaded onto the urea-glycerol gels. The resolving gel consisted of 10% acrylamide, 40% glycerol, and the stacking gel of 5% acrylamide, 20% glycerol both in 25 mmol/L glycine, 20 mmol/L Tris, pH 8.6. The running buffer contained 122 mmol/L glycine, 20 mmol/L Tris, pH 8.6. Proteins were transferred to nitrocellulose membranes. The membranes were blocked in 5% nonfat milk in TBS-Tween and incubated overnight with anti-RLC (cardiac isoform) primary antibody (a most generous gift from Prof. James Stull from UT Southwestern). Protein levels were detected by enhanced chemiluminescence using Amersham ECL Plus kit (GE Helathcare Life Sciences, Buckinghamshire, England) and Fujifilm LAS-3000 Imager (Fuji Photo Film Co., Tokyo, Japan).

### Western Blot Analysis for MAPK Phosphorylation

Frozen LV tissues were grinded in liquid nitrogen and were dissolved and homogenized in ice-cold lysis buffer containing of 20 mmol/L Tris, (pH 7.5), 10 mmol/L NaCl, 1 mmol/L EDTA, 1 mmol/L EGTA, supplemented with 1 mmol/L β-glycerophosphate, 2 mmol/L dithiothreitol, 1 mmol/L Na_3_VO_4_, 10 μg/mL leupeptin, 10 μg/mL aprotinin, 2 μg/mL pepstatin, 2 mmol/L benzamidine, 1 mmol/L phenylmethylsulfonyl fluoride and 20 mmol/L NaF. Samples were then centrifuged at 1300×g for 5 min at 4°C and the supernatant was collected. Protein concentrations were determined by the method of Bradford. Protein extracts were matched for protein concentration and stored denaturated in SDS loading buffer at −70°C. Equal volumes (30 μg) of protein samples were loaded onto 10% SDS-PAGE and transferred to nitrocellulose membranes. Protein levels were detected using fluorescence as described above. Quantification of the blots was done by using the Quantity One Basic 1-D Analysis Software (Bio-Rad Laboratories, Hercules, CA, USA). The antibodies used were anti-phospho-ERK1/2, anti-ERK1/2, anti-p38, and anti-phospho-p38 (Cell Signaling Technology Inc., Danvers, MA, USA).

### Statistical Analysis

SPSS Statistics 20 program was used for statistical analysis. Results are presented as mean±SEM. Repeated-measures ANOVA test was used to evaluate the statistical significance of differences among groups for cardiac contractility. The two factors were treatment group (with 4 categories: apelin, apelin+inhibitor, inhibitor, vehicle) and time as the repeated measure. When significant differences were detected in 2-way repeated measures ANOVA for the treatment-by-time interactions, a Bonferroni post hoc test was used for specific comparisons. 2 groups at a time were compared using unpaired Student’s t test; all other parameters were analyzed with 1-way ANOVA followed by Bonferroni post hoc test. Differences were considered statistically significant at the level of *P*<0.05.

## Results

### PKCε Is Involved in Apelin-Induced Inotropic Response

**Figure 1:**
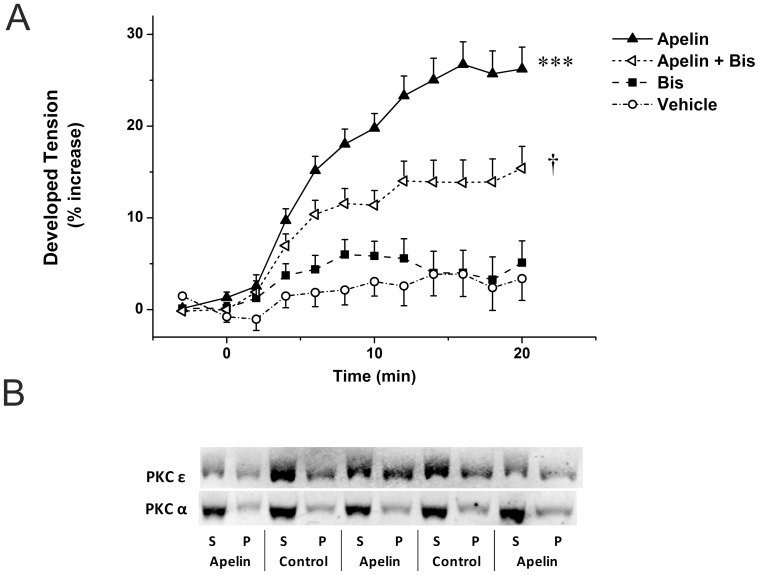
Positive inotropic effect of apelin mediated through PKC. A. Effect of apelin (Apelin-16 2 nmol/L) and Bis (90 nmol/L) on developed tension in isolated perfused, paced rat hearts. After a control period vehicle or drugs were infused for 20 minutes. Results are expressed as a percent change vs. baseline values. Data are mean ± SEM (n = 5). ***p<0.001 vs. vehicle control, †p<0.05 vs. apelin by repeated measures ANOVA and Bonferroni post-hoc test. **B.** Representative Western blot detection of translocation of PKCε and α isoforms from the soluble (S) to the particulate (P) fraction of left ventricular proteins in apelin treated and control animals.

In the isolated perfused rat heart preparation, administration of apelin (2 nmol/L) for 20 min induced a slowly developing and sustained increase in cardiac contractility (27±3%, *P*<0.001; Fig. 1A), in line with our former results demonstrating that this apelin isoform has a pronounced inotropic effect in the range of 0.1–10 nmol/L concentration [15]. To test the effect of different apelin isoforms on cardiac contractility, we performed pilot experiments with [Pyr1]apelin-13, apelin-13 and apelin-16, and found that the positive inotropic responses to these isoforms were on the same magnitude in our model (at 2 and 10 nmol/L concentrations, data not shown). These data are in agreement with the findings of Maguire and co-workers demonstrating that [Pyr^1^]apelin-13, apelin-13 and apelin-36 have comparable potency and efficacy in inducing positive inotropic effect in human paced atrial strips [12].

Our former experiments suggested that apelin may act via the PLC–PKC cascade [15]. In line with this, infusion of Bis (90 nmol/L), a selective PKC inhibitor, decreased the apelin-induced inotropic response by 42% (*P*<0.05; Fig. 1A). Infusion of Bis alone had no effect on contractile force (*P* = 1.0 vs. vehicle; Fig. 1A).

To provide further evidence that PKC contributed to apelin signaling, we examined the activation of PKCα and PKCε, the isoforms that are most important to the regulation of cardiac contractility [31], [32]. PKC isoforms show rapid translocation from the soluble to the particulate fraction of the cardiomyocyte upon stimulation [33]. When compared to controls, apelin treatment for 5 min produced a significant increase in the particulate partitioning of PKCε in the adult rat LV (Fig. 1B). However, during a more prolonged, 10-min apelin infusion, the subcellular distribution of PKCε returned to those in control hearts (data not shown), suggesting a transient increase in PKCε activation. In contrast to PKCε, no consistent PKCα translocation could be detected upon apelin administration (Fig. 1B and data not shown).

### Apelin Increases Cardiac Contractility via MLCK Activation

**Figure 2:**
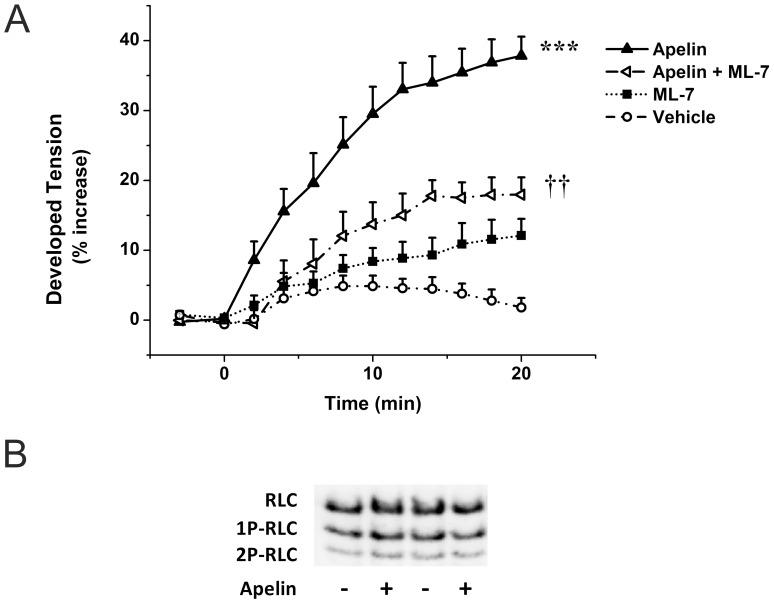
Positive inotropic effect of apelin mediated through RLC. A. Effect of apelin (Apelin-16 2 nmol/L) and ML-7 (1 μmol/L) on developed tension in isolated perfused, paced rat hearts. After a control period vehicle or drugs were infused for 20 minutes. Results are expressed as a percent change vs. baseline values. Data are mean ± SEM (n = 5). ***p<0.001 vs. vehicle control, ††p<0.01 vs. apelin by repeated measures ANOVA and Bonferroni post-hoc test. **B.** Representative Western blot detection of RLC phosphorylation in left ventricular proteins of apelin treated and control animals using non-denaturating urea gel electrophoresis. RLC: non-phosphorylated RLC; 1P-RLC: 1x phosphorylated RLC; 2P-RLC: 2x phosphorylated RLC.

Our previous findings suggest that apelin exerts its positive inotropic effect primarily through increasing the sensitivity of myofilaments to Ca^2+^ rather than increasing intracellular Ca^2+^ concentrations [17]. Increased phosphorylation of RLC by MLCK leads to an increase in the Ca^2+^ sensitivity of force development and improved cross-bridge kinetics in cardiac myofibrils [34]. To examine whether MLCK contributes to the positive inotropic effect of apelin, we used ML-7, a potent and selective inhibitor of MLCK, in the perfused adult rat heart. ML-7 (1 μmol/L) significantly attenuated the inotropic response to apelin, the maximal reduction being 52.5% (*P*<0.01). Infusion of ML-7 alone had no significant effect on contractile force when compared to vehicle control (*P* = 1.0; Fig. 2A).

Next, we performed urea-glycerol PAGE to separate phosphorylated and nonphosphorylated RLC in the apelin treated rat LV myocardium. The level of basal RLC phosphorylation was found to be comparable to the results presented by others using the same technique [24], [30], but apelin treatment failed to induce detectable increase in RLC phosphorylation under our experimental conditions (Fig. 2B).

### MAPK Signaling Contributes to Apelin-Induced Inotropic Response

**Figure 3:**
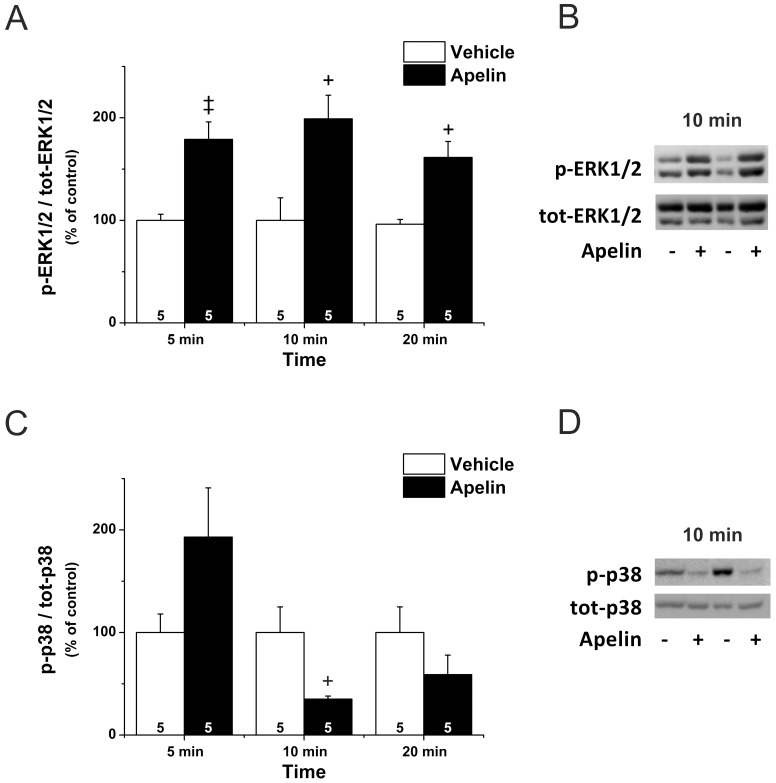
Apelin and MAPK signaling. A. Western blot analysis of time-dependent ERK1/2 phosphorylation in left ventricles. Results in the bar graph are expressed as the ratio of phospho-ERK1/2 (p-ERK 1/2) and total (tot) ERK1/2 in percent of values in vehicle-treated control animals (n = 5–6). **B.** ERK1/2 phosphorylation in a representative blot of left ventricular proteins from hearts treated with apelin or vehicle for 10 minutes. **C.** Western blot analysis of time-dependent p38 phosphorylation in left ventricles. Results in the bar graph are expressed as the ratio of phospho-p38 (p-p38) and total (tot) p38 in percent of values in vehicle-treated control animals (n = 4–5). **D.** p38 phosphorylation in a representative blot of left ventricular proteins from hearts treated with apelin or vehicle for 10 minutes. **+**P<0.05 vs. vehicle, ‡ P<0.01 vs. vehicle by unpaired Student’s t test.

To explore the potential involvement of MAPK signaling in modulating the inotropic response to apelin, we assessed the apelin-induced changes in ERK1/2 and p38-MAPK phosphorylation. Immunoblotting revealed that apelin induced a sustained increase in LV ERK1/2 phosphorylation (P<0.01 at 5 min, *P*<0.05 at 10 and 20 min vs. controls), with a maximum increase of 99±23% at 10 min (Fig. 3A and 2B). Phosphorylation of p38-MAPK showed a clear but non-significant trend for an increase after 5 min. On the contrary, by 10 min of infusion, apelin significantly decreased p38-MAPK phosphorylation (−65±3% vs. control, *P*<0.05; Fig. 3C–D).

**Figure 4:**
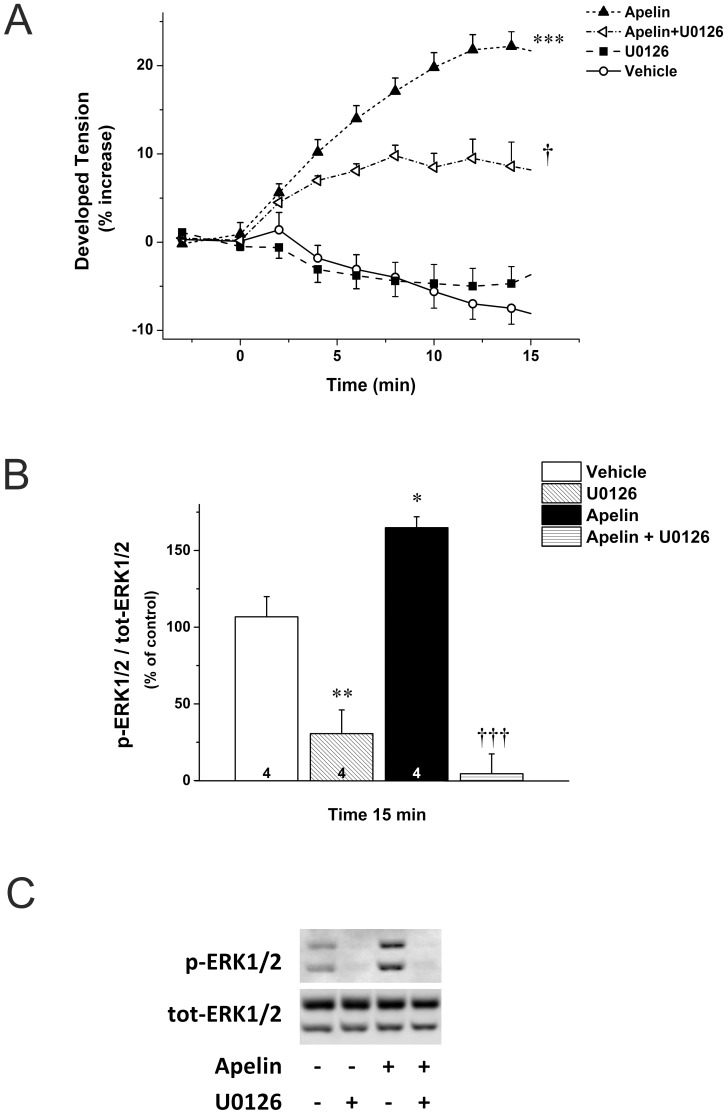
Positive inotropic effect of apelin mediated through ERK1/2. A. Effect of apelin (Apelin-16 12 nmol/L) and U0126 (5 μmol/L) on developed tension in isolated perfused, paced rat hearts. After a control period vehicle or drugs were infused for 15 minutes. Results are expressed as a percent change vs. baseline values. Data are mean ± SEM (n = 4). **B.** Western blot analysis of ERK 1/2 phosphorylation in left ventricles of hearts treated with vehicle, apelin and U0126, or their combination for 15 minutes. Results in the bar graph are expressed as the ratio of phospho-ERK1/2 (p-ERK 1/2) and total (tot) ERK1/2 (n = 5). **C.** ERK1/2 phosphorylation in a representative blot of left ventricular proteins from hearts treated with vehicle, apelin and U126, or their combination for 15 minutes. *P<0.5; **P<0.01; ***P<0.001 vs. vehicle, †P<0.5; †††P<0.001 vs. apelin by repeated measures ANOVA and Bonferroni post-hoc test.

To demonstrate that ERK1/2 activation is necessary to the development of apelin-induced inotropic response, we used U0126, which is a potent selective inhibitor of MAPK kinases 1 and 2 (MEK1/2), the upstream regulator of ERK1/2. The inotropic effect of apelin was significantly attenuated by U0126 (5 μmol/L), the maximal reduction being 56% (P<0.05). Infusion of U0126 alone had no significant effect on contractile force (*P* = 1.0; Fig. 4A). Immunoblotting of LV lysates showed that U0126 almost completely abolished ERK1/2 phosphorylation after 15 min of perfusion, either administered alone (31±15% of control, *P*<0.01) or in combination with apelin (4±8% of the apelin-treated group, *P*<0.001; Fig. 4B–C).

**Figure 5:**
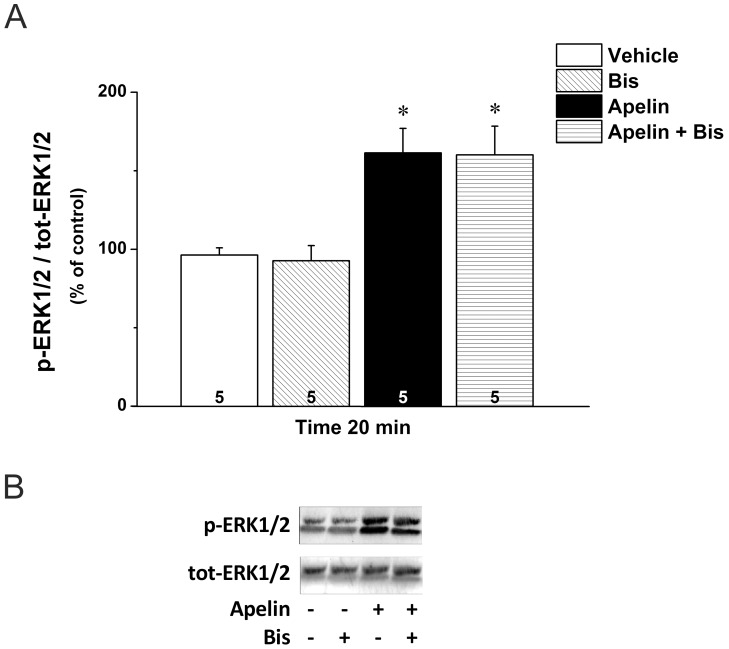
Relation of apelin-induced ERK1/2 phosphorylation to PKCs. A. Western blot analysis of ERK 1/2 phosphorylation in left ventricles of hearts treated with vehicle, apelin and Bis, or their combination for 20 minutes. Results in the bar graph are expressed as the ratio of phospho-ERK1/2 (p-ERK 1/2) and total (tot) ERK1/2 (n = 5). *p<0.05 vs. vehicle by one-way ANOVA followed by a Bonferroni post hoc test. **B.** ERK1/2 phosphorylation in a representative blot of left ventricular proteins from hearts treated with vehicle, apelin and Bis, or their combination for 20 minutes.

Particulate partitioning of PKCε in neonatal rat ventricular myocytes is accompanied by subsequent activation of ERK1/2 [35]. Since apelin significantly increased PKCε translocation and ERK1/2 phosphorylation in the intact rat heart, we examined whether PKC is an upstream activator of ERK1/2 in apelin signaling. Interestingly, we found that the PKC inhibitor Bis, which potently attenuated the apelin-enhanced contractility, had no effect on the apelin-induced increase in ERK1/2 phosphorylation (Fig. 5A–B), demonstrating that ERK1/2 and PKC represent independent pathways mediating the inotropic effect of apelin.

## Discussion

Apelin is among the most potent endogenous inotropes yet identified [15]; however, the cellular mechanisms underlying its inotropic effect are not fully clarified. This study provides several important findings regarding the signaling network activated by apelin in the adult rat heart. The present results demonstrate that pharmacological inhibition of PKC significantly reduces the positive inotropic effect of apelin, confirming previous data from our [15] and other laboratories [36]. The PKC family consists of a variety of isoenzymes, e.g. classical (α, βI, βII, and γ), novel (δ, ε, θ, and η) and atypical PKCs (ζ, ι/λ). Individual isoenzymes can have different, even opposing functions [37] and they are each localized to distinct subcellular sites following activation [38]. Various PKC isoforms are considered to regulate cardiac contractility [31], [32]. However, the exact PKC isoenzyme that contributes to the apelin-induced contractile response has not been identified yet. Our present data indicate that apelin promotes PKCε but not PKCα translocation to the particulate fraction. Specific PKCε anchoring proteins are localized at the Z-lines and intercalated discs in cardiomyocyte [39]. Upon activation, PKCε is known to accumulate in these very specific regions of ventricular myocytes, resulting in a strong positive inotropic effect [40]. These findings locate activated PKCε to the close vicinity of apelin receptor, the cognate receptor of apelin [17].

RLC controls myofilament cross-bridge properties and thereby modulates the force of contractions in the heart. RLC is phosphorylated by the cardiac MLCK [24] which is counterbalanced by the activity of myosin light chain phosphatase [41]. Increased RLC phosphorylation results in an increase of the Ca^2+^ sensitivity of myofilaments [34]. The phosphate turnover rate of cardiac RLC is much slower than that of skeletal or smooth muscle cells, suggesting that cardiac RLC plays a sustained, fine-tuning role in adjusting the kinetic properties of the contractions [42]. Since the force development in response to apelin is comparable in timescale to that of RLC phosphorylation in the heart, one may speculate that apelin improves myofilament function through activation of MLCK. In line with that, we demonstrate here that MLCK inhibition diminishes the apelin-enhanced contractility. The pharmacological inhibitor used here is known to act with high selectivity on MLCK, an enzyme that functions solely as a kinase for RLC. It has been reported that ML-7 inhibits sarcomeric organization in rat cardiomyocytes in a similar fashion to cardiac MLCK RNA interference [43] and reduces RLC phosphorylation in isolated rat ventricular strips [44]. The 1 μmol/L ML-7 dose applied in our experiments provide even higher selectivity to MLCK than the 10 or 20 μmol/L doses used by the above mentioned studies, based on the Kinase Inhibitor Database of the MRC Protein Phosphorylation Unit at Dundee (http://www.kinase-screen.mrc.ac.uk/kinase-inhibitors). Therefore it is plausible to assume that the apelin-mediated increase in cardiac contractility is partly dependent on MLCK activation. Nevertheless, no significant apelin-induced increase in RLC phosphorylation was detected by urea-glycerol PAGE. One should consider, however, that given the rate of approximately 40% of RLC phosphorylation under basal physiological conditions [42], only modest increase in phosphorylation is conceivable. Still, a subtle change can be sufficient to have a significant effect on contractility. It was demonstrated in isolated rat papillary muscles that even a less than 10% increase in the overall RLC phosphorylation level can be attributed to a 70% increase in contractile force [44]. One limitation of the current study is that such small changes may remain undetectable in the intact heart under our *ex vivo* experimental conditions.

The exact mechanisms of cardiac MLCK activation remain elusive. Contrasting smooth- and skeletal muscle isoforms, cardiac MLCK was found to be Ca^2+^/calmodulin-independent. On the other hand, potential phosphorylation sites for PKC were identified on cardiac MLCK [45]. Some studies demonstrated PKC-dependent RLC phosphorylation in the heart [23], [46], but others provided evidence challenging the role of PKC in triggering RLC regulation [47], [48]. Although MLCK is controlled remarkably differently in cardiac and smooth muscle tissues, it is noteworthy that the inhibition of PKC markedly attenuated the apelin-induced RLC phosphorylation in vascular smooth muscle cells [25]. Therefore, cardiac MLCK and RLC are potential downstream targets of PKC, mediating apelin-triggered positive inotropic response.

The MAPKs are well known regulators of diverse processes in the heart under physiological and pathophysiological conditions [49], but only a few reports demonstrated that MAPKs can regulate cardiac contractility [26], [50]. Our study provides evidence that apelin activates ERK1/2 in the myocardium, and suppression of ERK1/2 signaling significantly attenuates the apelin-mediated increase in the contractile force. Previously we have demonstrated that activation of NHE contributes to the inotropic effect of apelin [15], [17]. Since ERK1/2 is a recognized activator of NHE [51], we propose here a functional ERK1/2-NHE axis in apelin signaling. ERK1/2 can be activated, among many others, by PKCs [35]. Knowing that PKC is involved in the inotropic effect of apelin, one can speculate that PKC is an upstream regulator of ERK1/2. Our finding, that PKC inhibition, which is sufficient to reduce the inotropic response to apelin, does not decrease apelin-induced ERK1/2 phosphorylation indicates that apelin activates ERK1/2 via a PKC-independent mechanism. Of note, approximately 50% of the inotropic response to apelin remained unaffected even if ERK1/2 phosphorylation was practically undetectable. Thus, PKC and ERK1/2 are parallel and independent signaling pathways mediating the effect of apelin on cardiac contractility. In contrast to ERK1/2, apelin significantly reduced p38-MAPK phosphorylation in the intact rat heart. Activation of p38-MAPK appears to have an important homeostatic function by counterbalancing excess inotropic stimulation. β_2_-adrenergic receptor or endothelin receptor stimulation increases p38-MAPK activation, and pharmacological inhibition of p38-MAPK activation augments β_2_-adrenergic receptor - or endothelin-mediated increases in cardiac contractility [26], [52]. Moreover, p38-MAPK activation has a crucial role in delivering the negative inotropic effect of tumor necrosis factor-α [53]. However, the impact of reduced p38-MAPK activity on cardiac contractility is controversial. Some studies suggest that pharmacological [26] or genetic inactivation of p38-MAPK [50] or its upstream kinase MKK3 [53] alone had no effect on baseline cardiac contractility, whereas others propose that reducing p38-MAPK activity by chemical or genetic approaches may indeed augment contractile force [50], [54]. Whether the observed decrease in p38-MAPK phosphorylation may contribute to the apelin-mediated increase in cardiac contractility remains to be defined.

Accumulating lines of evidence suggest that the activation of PKCε and MEK1/2–ERK1/2 cascades constitute important adaptive mechanisms in the myocardium under pathological conditions. PKCε and ERK1/2 signaling have been reported to confer cardioprotection in vivo against ischemia-reperfusion injury by reducing cell death [55], [56]. Using a genetic model, loss of apelin exacerbated myocardial ischemia-reperfusion injury associated with compromised activation of the MEK1/2–ERK1/2 signaling pathway [57]. In addition to regulating cell survival, PKCε and ERK1/2 also control the pattern of LV remodeling. Inhibition of PKCε translocation triggered LV enlargement and wall thinning with depressed contractile function in Gα_q_-overexpressing mice. Reciprocally, enhanced PKCε activation resulted in a more favorable LV geometry with improved LV performance in Gα_q_ mice, displaying concentric instead of eccentric remodeling [58]. Moreover, it has recently been demonstrated in genetically modified mice that the MEK1/2–ERK1/2 signaling pathway directly regulates the balance between eccentric and concentric growth of the heart. Constitutive ERK1/2 activation promotes concentric cardiomyocyte hypertrophy, whereas ERK1/2 deficiency leads to pronounced eccentric hypertrophy in response to increased mechanical load or neurohumoral stimulation [59]. Although the underlying signaling mechanisms remain to be explored, apelin deficiency in chronic pressure overload resulted in severe heart failure characterized by LV dilation and impaired cardiac performance [14]. Our current results raise the intriguing possibility that stimulation of the apelin–apelin receptor system with concomitant activation of ERK1/2 and PKCε signaling, besides inhibiting eccentric growth and apoptosis, may also alleviate hemodynamic stress in the injured heart by directly improving cardiac contractility.

## Conclusion

The present study provides evidence that apelin increases cardiac contractility through parallel and independent activation of PKCε and MEK1/2–ERK1/2 signaling in the adult rat heart. Moreover, MLCK activation represents a downstream mechanism by which apelin may increase Ca^2+^-sensitivity of the contractile machinery. Modest activation of PKCε and MEK1/2–ERK1/2 signaling have been reported to confer protection against stress-induced myocyte apoptosis [55], [56]. Signaling pathways that promote cardiomyocyte survival while improving contractile function may offer an attractive alternative in treating patients with heart failure.
